# Analog Synaptic Plasticity in 2D Layered Material Iontronic Memtransistors for Brain‐Inspired Computing

**DOI:** 10.1002/advs.77150

**Published:** 2026-08-18

**Authors:** Puranjay Saha, Saptarshi Bej, Bikas C. Das

**Affiliations:** ^1^ eNDR Laboratory School of Physics IISER Thiruvananthapuram Trivandrum Kerala India; ^2^ School of Data Science IISER Thiruvananthapuram Trivandrum Kerala India

**Keywords:** cognitive learning, iontronics, memtransistor, molybdenum disulfide, neuromorphic computing, synaptic Plasticity

## Abstract

A simple iontronic memtransistor capable of emulating synaptic plasticity and cognitive functions showcases high performance and low energy consumption, fostering admiration for its efficiency. Here, we report the versatile, high‐performance memtransistor behavior of a solid polymer electrolyte‐gated few‐layer thick two‐dimensional molybdenum disulfide (2D MoS_2_) channel. Transfer characteristics exhibit pinched hysteresis, confirming n‐channel enhancement‐mode operation, supported by the low‐voltage drain characteristics under the influence of the electrical double layer (EDL) formed by iontronic gating. The memtransistor shows a reproducible non‐volatile memory window in its transfer characteristics with conductance retention exceeding 10^3^ s and endurance beyond 10^3^ switching cycles. Mechanistic studies reveal coupled slow ion migration and dipolar relaxation processes coexisting with purely electronic transport in the 2D material channel, highlighting mixed ionic‐electronic carrier dynamics. Using tailored input‐output pulse schemes, the device demonstrates key synaptic and cognitive learning functionalities with lower energy consumption per event and faster response speed down to the microsecond regime. Furthermore, higher‐order behaviors such as Atkinson‐Shiffrin‐type memory behavior, Pavlovian associative learning, and logic gate operations are achieved. These achievements confirm the potential of our 2D iontronic memtransistor (IMT) as a reliable, reproducible building block for next‐generation brain‐inspired computing systems.

## Graphical abstract

In this work, we demonstrated a robust 2D MoS_2_ based iontronic memtransistor of planar architecture operated under the influence of an electrical double layer with versatile performance and applications, including pinched hysteresis nature of transfer curve, analogue channel conductance tuning, low‐voltage operation, logic‐gate operation, classical conditioning of the brain, and various synaptic and neural behaviors.

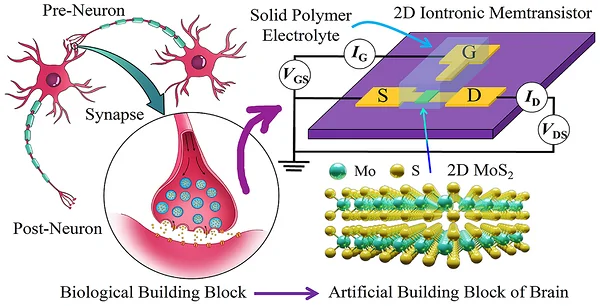

## 1 Introduction

Neuromorphic computing represents a fundamental reassessment of information processing, changing from algorithm‐driven architectures to systems in which learning, memorization, and adaptation emerge directly from device physics [1, 2]. Motivated by the human brain, this paradigm of simultaneous information storage and processing seeks to unify sensing, memory, and computation within the same physical elements, overcoming the fundamental issues with the energy, latency, and scalability limitations of von Neumann architectures [3, 4]. Central to this idea is the realization of artificial synapses of solid‐state electronic device that are not only programmable, but intrinsically adaptive, capable of generating multiscale plasticity, learning reinforcement, and memory consolidation [5]. Although memristors and memtransistors have enabled a significant progress for mimicking synaptic plasticity, but the most existing platforms remain constrained by inferior energy efficiency, stochastic behavior, limited analog conductance modulation, poor endurance, and lack of reproducibility, preventing reliable development toward efficient neuromorphic systems [6].

Solid‐state iontronic devices provide a biologically inspired route toward adaptive hardware by embedding ionic dynamics into electronic transport, thereby facilitating the emergence of learning and memory from coupled electrochemical‐electronic processes [7, 8]. Within this framework, two‐dimensional transition metal dichalcogenides (2D TMDs) arise as uniquely effective channel materials [9, 10, 11]. Their thickness down to the atomic level, clean van der Waals interfaces, strong gate electrostatic coupling, and high surface sensitivity allow external ionic double‐layer field, dipoles, and interfacial polarization to directly modulate channel conductance with superior efficiency and precision [12, 13]. Unlike bulk semiconductors, 2D TMDs naturally amplify interfacial phenomena due to their large surface area exposures, making them ideal transduction layers for iontronic control [14]. Moreover, their intrinsic stability, scalable fabrication compatibility, and tunable electronic structure make them robust platforms for hybrid ionic‐electronic neuromorphic architectures [15]. The challenge of iontronics in solid‐state devices is already enriched by various solid polymer electrolyte thin films as the gate dielectric, particularly a combination of lithium perchlorate (Li(ClO_4_)_2_) as the ionic counterpart within poly(ethylene oxide) (PEO) as the polymer matrix [16, 17, 18]. The confluence of iontronics with 2D electronics through solid ion‐conducting polymer electrolyte therefore demonstrates a fundamentally different device paradigm, in which adaptive learning, memorization, and adaptive learning behaviors emerge from physically coupled ionic and electronic degrees of freedom within an atomically thin crystalline channel.

In this work, we present an 2D iontronic memtransistor (2D‐IMT) based on mechanically exfoliated few‐layer thick molybdenum disulfide (MoS_2_) that integrates stable enhancement‐mode n‐channel operation with non‐volatile analogue memory and mixed ionic‐electronic transport dynamics. The 2D‐IMT devices depict reproducible memory windows, long retention of multiple analogue conductance states, high endurance, microsecond‐scale response speeds, and ultra‐low energy consumption. Impedance spectroscopy and Raman scattering reveal coupled slow ion migration and dipolar relaxation interacting with purely electronic transport in the 2D MoS_2_ channel, establishing the physical origin of the observed plasticity. This controlled ion‐electron coupling enables faithful emulation of synaptic functions, including excitatory and inhibitory postsynaptic currents, paired‐pulse facilitation and depression, Hebbian learning rules, and memorization models, as well as training‐induced plasticity and classical conditioning through programmable pulse schemes. Beyond synaptic emulation, experimentally recorded synaptic weight dynamics are directly integrated into a hybrid spiking‐convolutional neural network framework to achieve efficient pattern recognition on the MNIST dataset, demonstrating the transition from device‐level plasticity to system‐level classical conditioning of brain. Together, this architecture establishes iontronic 2D memtransistors based on TMD channels as scalable, physically grounded building blocks for neuromorphic and cognitive computing platforms.

## 2 Results and Discussion

### 2.1 Iontronic 2D MoS_2_ Memtransistor Device Performance

To realize high‐performance 2D iontronic memtransistors (2D‐IMTs), few‐layer molybdenum disulfide (MoS_2_) flakes were mechanically exfoliated using a Gel‐Pak elastomeric stamp and transferred onto Si/SiO_2_ substrates bearing pre‐patterned Au source‐drain electrodes. A laterally isolated gate electrode was defined on the same substrate, forming a planar side‐gated architecture. Figure 1a schematically illustrates the trigonal prismatic 2H crystalline phase of MoS_2_, in which a hexagonal molybdenum layer is sandwiched between two sulfur layers, forming covalently bonded S‐Mo‐S units stacked via weak van der Waals interactions. This layered structure facilitates mechanical exfoliation while preserving intrinsic semiconducting properties. Atomic force microscopy (AFM) topography images (Figure 1b and Figure S1) indicate an average flake thickness of ∼5 nm, corresponding to approximately 6 – 7 layers of MoS_2_ [19, 20]. Raman scattering result affirms the characteristic vibrational modes of 2H‐MoS_2_, and photoluminescence measurements confirm its semiconducting nature (Figure S2), consistent with a few‐layer MoS_2_ flake [15].

**FIGURE 1:**
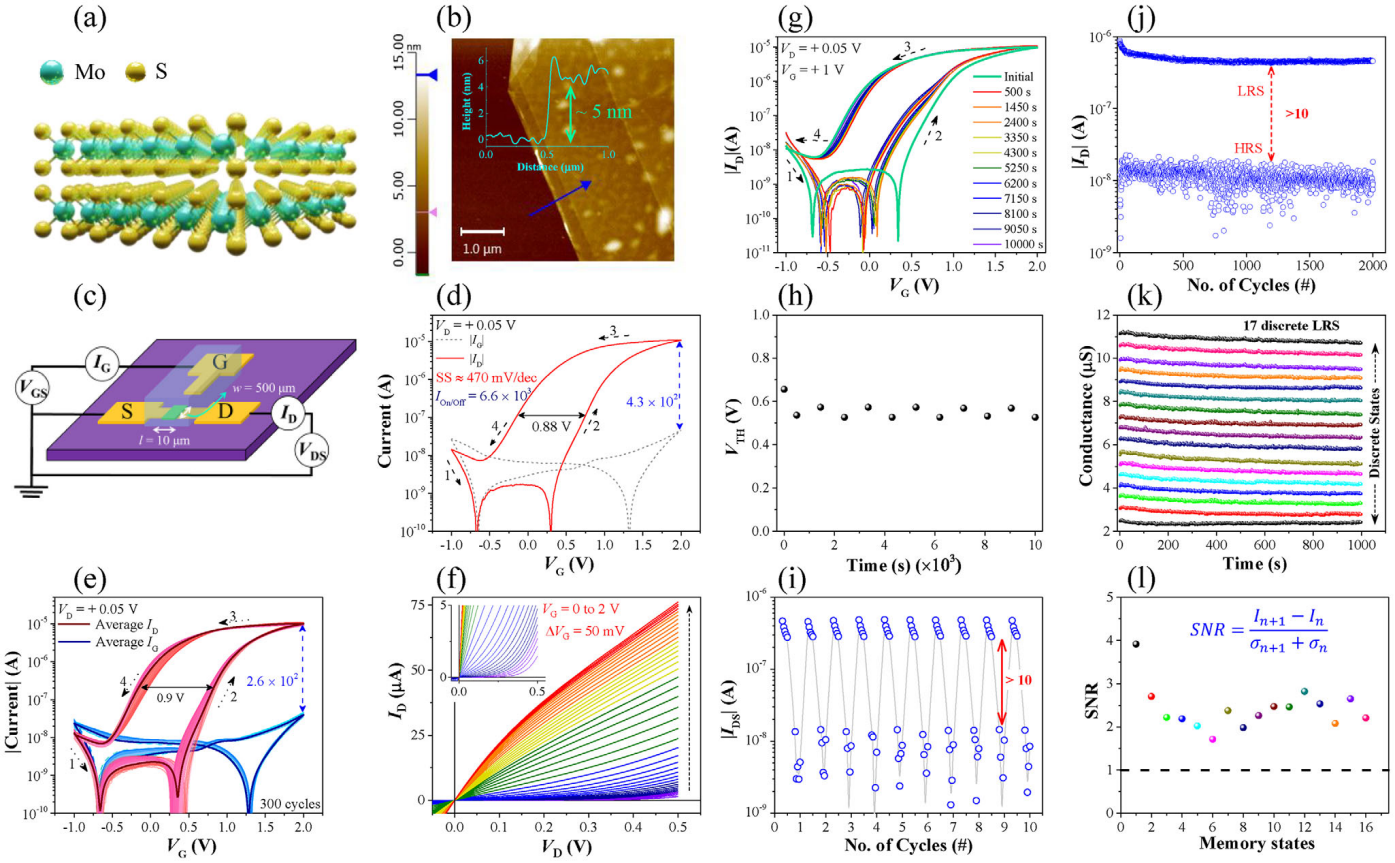
(a) The atomic structure of layered 2H‐MoS_2_. (b) Atomic force microscope (AFM) image of mechanically exfoliated few‐layered 2D MoS_2_ with an overlaid height profile indicating thickness of ∼ 5 nm extracted along the marked blue arrow line. (c) Device schematics for electrical characterizations. (d) A transfer curve (*I*_D_ vs *V*_G_, solid red line) recorded in a loop by sweeping *V*_G_ between +2 and −1 V at a fixed drain voltage bias (*V*_D_ = +0.05 V). The simultaneously recorded iontronic gate current (*I*_G_ vs *V*_G_, dashed gray line) is also included. (e) Continuously recorded 300 cycles of transfer curves and the corresponding gate current reflecting excellent performance stability. (f) Output characteristics (*I*_D_ vs *V*_D_) recorded by sweeping *V*_D_ between −0.1 and +0.5 V and varying *V*_G_ from 0 to +3 V in steps of 50 mV. (g, h) Positive bias stress (PBS) test by recording transfer curves at a regular time intervals over a period of 10^4^ s and the corresponding threshold voltage (*V*_TH_) variation during the PBS test. (i) Resistive random access memory (ReRAM) behavior for 10 W/R/E/R cycles. (j) Endurance recorded over 2 × 10^3^ cycles. (k) Retention of 17 different low resistive states for more than 10^3^ s. (l) Signal‐to‐noise ratio (SNR) extracted for all the conducting states pairs produced in (k) displaying values well above the unity threshold.

These 2D MoS_2_ flakes were used as the active channel for the fabrication of 2D‐IMT devices. Iontronic gating was achieved by depositing a solid polymer electrolyte (SPE) composed of LiClO_4_:PEO (1:9 w/w), which covered both the channel and the gate electrode (Figure 1c; Methods). A representative optical micrograph image of a final device is shown in Figure S3. Notably, the fabricated devices have a channel length of about 10 µm and a width of about 500 µm, as labelled in Figure 1c. Device integrity was systematically assessed by measuring current–voltage (*I–V*) characteristics between source‐drain (S‐D) and source‐gate (S‐G) terminals before and after depositing the electrolyte layer (Note S1). After SPE integration, a noticeable increase in S‐D current is observed under iontronic electrolyte gating, while the S‐G mixed ionic‐electronic current remains several orders of magnitude lower than the channel conductance, corroborating adequate ion‐mediated gating without electrical leakage (Figure S4) [21, 22]. Compared with traditional top‐gated SPE thin‐film transistor architectures, the coplanar side‐gated electrode configuration ameliorates leakage pathways and mitigates parasitic capacitance effects [23]. The resulting 2D‐IMT device architecture offers several practical advantages, including simplified fabrication steps, cost‐effectiveness, compatibility with scalable lithographic processes, and potential integration with complementary metal‐oxide‐semiconductor (CMOS) platforms. This utilization of the materials‐to‐device fabrication procedure establishes a robust foundation for exploring iontronic modulation of MT channel conductance analogously in atomically thin layered semiconductors.

To probe the effective electrochemical gating behavior of our 2D‐IMT devices, we first diagnosed their transfer characteristics by recording the drain current (*I*_D_) as a function of gate voltage (*V*_G_), keeping the drain bias constant (*V*_D_ = +0.05 V). The gate voltage was swept in loops between –1.0 and +2.0 V. A representative transfer characteristic (Figure 1d), recorded simultaneously with the gate current (*I*_G_), portrays a clear counter‐clockwise hysteresis in log‐linear scale with a memory window of ∼0.88 V between forward and reverse scans, as a signature of non‐volatile resistive switching (NV‐RS) in 2D‐IMT devices [22, 24]. The devices demonstrate a subthreshold swing of 470 mV/dec, an on/off ratio of ∼10^4^, and a conductivity contrast surpassing two orders of magnitude between drain and gate currents, reflecting effective ionic‐electronic coupling [7]. The hysteresis in the transfer characteristic is found to be highly reproducible over 300 consecutive cycles (Figure 1e), evidencing stable electrochemical or iontronic modulation of channel conductivity without noticeable degradation. The output characteristics further disclose gradual and analogue conductance modulation upon incremental increases of *V*_G_ in steps of 50 mV starting from 0 to +3.0 V (Figure 1f) [25]. Such a finely tunable channel conductance modulation arises from progressive ionic redistribution in the solid electrolyte layer and supports multi‐level conductance states desirable for synaptic weight encoding. Together, the transfer and output characteristics of our 2D‐IMT devices confirm n‐channel enhancement‐mode operation.

Device performance parameters (Figure S5) extracted from the linear region of the transfer curve (Note S2) yield a threshold voltage (*V*_TH_) of 0.78 V, a maximum transconductance (*g*_m_) of ∼10 µS, and a field‐effect mobility (µ_FE_) of 0.23 cm^2^V^−1^s^−1^. These values are found to be significant for solid electrolyte‐induced high‐capacitance gating in a two‐dimensional TMD‐based MT device. To evaluate long‐term electrochemical stability, we conducted bias‐stress measurements by continuously applying *V*_G_ = +1.0 V for up to 10^4^ s and recording transfer curves at *V*_D_ = +0.05 V (Figure 1g). The transfer characteristics remain largely invariant throughout the stress period, with an average *V*_TH_ of 0.54 V after the initial sweep (0.65 V), indicating minimal threshold drift and robust ion‐mediated channel modulation (Figure 1h). In view of the robust and stable device operation, we systematically quantified both cycle‐to‐cycle and device‐to‐device variability across key performance metrics (Note S3). The statistical distributions of *V*_TH_ and subthreshold swing (*SS*) were extracted from 300 consecutive resistive switching cycles in Figure 1e as well as from devices fabricated across six independent batches (Figure S6). These analysis reveal narrow parameter dispersion (Figure S7). Cumulative probability plots and Weibull statistics of the high‐resistance state (HRS) and low‐resistance state (LRS) (Figure S8) further confirm a resistance separation of ∼10^4^ between the two states. Together, these results highlight the reproducibility of the iontronic gating process of our 2D IMT devices. To further assess operational resilience, we fabricated additional 2D IMT devices using a higher ionic salt concentration (1:4 w/w ratio) in the solid polymer electrolyte (Note S4). These devices exhibit highly stable hysteretic transfer characteristics over ∼250 switching cycles, along with analogous channel conductance modulation and sustained bias stability (Figure S9). Although a slightly elevated high‐resistance state (HRS) is observed, the overall NV‐RS behavior remains robust. These results demonstrate that the operation of the 2D IMTs can be systematically tuned through the ionic salt concentration in the electrolyte, underscoring the adaptability and stability of the iontronic gating mechanism.

Having established the NV‐RS behavior, we systematically examined its bias dependence by recording transfer characteristics at varying drain voltages (*V*_D_) and gate‐voltage (*V*_G_) sweep ranges (Figure S10a,b). The memory window observed in the transfer curve widens markedly with higher + *V*_D_ applied and extended positive *V*_G_ sweep limits, implying enhanced ionic redistribution and stabilization of interfacial charge carriers under stronger electrochemical driving forces. The non‐volatile nature of the 2D‐IMTs facilitates resistive random‐access memory (ReRAM) functionality. Figure 1i exhibits read current response recorded by applying a constant *V*_D_ = +50 mV at the drain, while write (W) and erase (E) gate voltage (*V*_G_) pulses are applied at the gate alternatively. The device exhibits reproducible switching between low‐resistance and high‐resistance states (LRS and HRS) with an on/off ratio exceeding 10. Endurance measurements over 2 × 10^3^ cycles (Figure 1j) demonstrate stable data storage operation for ReRAM application, confirming reliable non‐volatile data storage. In addition to bistable states, our 2D‐IMT devices support analogue modulation of channel conductance with long‐term retention. By applying gate voltage (*V*_G_) pulses of increasing numbers (Figure S11a) and monitoring the drain conductance for up to 10^3^ s (Figure 1k), we resolved 17 distinct and stable conductance levels with no signature of mixing between two consecutive states. Signal‐to‐noise ratio (SNR) analysis (Note S5) for each state pair yields values exceeding unity (Figure 1l), validating the integrity of multilevel storage. Such multi‐bit programmability is promising for synaptic weight encoding in neuromorphic hardware architectures. Our 2D‐IMT devices also exhibit a response time of ∼1 µs (Figure S12), supporting low‐energy operation down to a few hundred femtojoules per event. Moreover, programmable gate biases enable implementation of OR and AND logic functions (Note S6) through direct current readout at the drain (Figure S13). To emphasize the benchmark achievements made in this work, Table 1 summarizes key performance metrics alongside the existing literature on similar‐type devices using 2D MoS_2_ as the active channel and a variety of iontronic gate dielectrics, including solid polymer electrolytes. Together, the fast switching dynamics, multilevel non‐volatility, and logic functionality highlight the potential of these 2D IMTs as compact building blocks for energy‐efficient neuromorphic hardware.

**TABLE 1:** Summary of key performance metrics of our iontronic memtransistor device, comparing with the already reported articles of devices mostly using side‐gated architectures and mechanically exfoliated 2D MoS_2_, if not highlighted specifically.

Dielectrics	Operating Voltage	*I*_ON_ */ I*_OFF_	Response Speed	Energy / Operation	Endurance/ Retention	Operating Mode	Mechanism	Cognition/ Pattern Recognition
[26] PVA	±1.5 V	>10^4^	10 ms	23.6 pJ	—	n‐channel	Carrier trapping /de‐trapping	No/No
[27]PVA	±1.5 V	>10^4^	—	—	—	—	Carrier trapping /de‐trapping	No/No
[16]PEO: LiClO_4_	+3 V/ −7 V	>10^5^	—	—	—	—	Redox reaction	No/No
[17]PEO: LiClO_4_	±1 V	>10^5^	—	—	—	n‐channel	Ionic diffusion	No/No
[28] Chitosan polymer	±1.5 V	>10^4^	10 ms	5 pJ	—	n‐channel	Proton‐electron coupling	No/No
[29]LiClO_4_: Chitosan	±1.5 V	>10^6^	—	17.5 nJ	—	n‐channel	Carrier trapping/ de‐trapping	No/No
[18]PEO: LiClO_4_ ^a^	±5 V	—	50 ms	500 pJ	—	n‐channel	Ionic diffusion	No/No
[30]LiSiO_x_ ^b^	±5 V	—	1 s	0.55 nJ	—	n‐channel	Electro‐chemical doping	No/Yes
^c^PEO: LiClO_4_	−1 V/ +2 V	∼10^4^	1 µs	280 fJ	2000 cycles/ 1000 s (4 bit)	n‐channel in enhancement mode	Reversible growth & decay of EDL	Yes/Yes

^a^ Vertical architecture.

^b^ Vertical architecture & PECVD grown MoS_2_.

^c^ This work.

### 2.2 Elucidating Coupled Electron‐Ion Carrier Dynamics

Lithium ions are often expected to intercalate into layered 2D MoS_2_, potentially inducing a phase transition to the metallic 1T′ polymorph [8]. To determine whether such structural transformation occurs during device programming, we performed in situ Raman spectroscopy under different applied gate bias voltages (Figure 2a). The spectra show no signatures characteristic of the 1T′ phase (Figure S14), indicating that Li^+^ ions do not intercalate into the MoS_2_ lattice under operating conditions [31]. Instead, a slight blue shift of the Raman modes is observed upon applied gate biasing, attributable to restrict on in‐plane and out‐of‐plane scattering modes. The observed gate‐dependent modulation of Raman peak intensities further suggests electrostatic and ion‐induced carrier‐density tuning into the 2D MoS_2_ channel rather than phase transition [32]. To probe the dielectric response of the PEO:LiClO_4_ electrolyte and the MoS_2_/SPE interface, we carried out electrochemical impedance spectroscopy (EIS). Bode (Figure 2b,c) and Nyquist plots (Supporting Information Figure S15) measured between the source‐gate (S‐G) terminals in both the HRS and LRS reveal three frequency regimes consistent with an equivalent R‐C circuit (inset of Figure 2b). At high frequency, a near‐capacitive response reflects restricted Li^+^ motion. An intermediate regime marks the onset of ionic diffusion and dipolar relaxation, while the low‐frequency response is dominated by electric double‐layer (EDL) formation at the MoS_2_/SPE interface [33, 34]. The data are well described by a constant phase element (Q) associated with EDL growth and a Warburg (W) component representing ionic diffusion in SPE layer [22]. The LRS exhibits reduced impedance relative to the HRS, consistent with enhanced interfacial charge accumulation across the channel between S‐D. The operating mechanism is therefore governed by reversible Li^+^ accumulation at the MoS_2_ surface under positive gate bias, forming a high‐capacitance EDL that enhances channel conductance (Figure 2d–g). Partial ion back‐diffusion upon bias removal enables conductance retention, while negative gate biasing voltage restores the pristine state without structural phase transformation [35]. The SPE exhibits excellent dielectric characteristics, with a steady‐state dielectric constant (ε ≈ 145), dielectric loss (tan δ ≈ 0.2), and capacitance of 0.86 nF measured at ∼1 kHz (Figure S16). Further, the R‐C equivalent circuit analysis from the measured EIS data yields characteristic relaxation times of 0.61 and 19.7 ms for the HRS and 0.44 and 20 ms for the LRS. These times corresponds to the fast interfacial charge accumulation and slower electric dipole relaxation in the solid polymer electrolyte, respectively.

**FIGURE 2:**
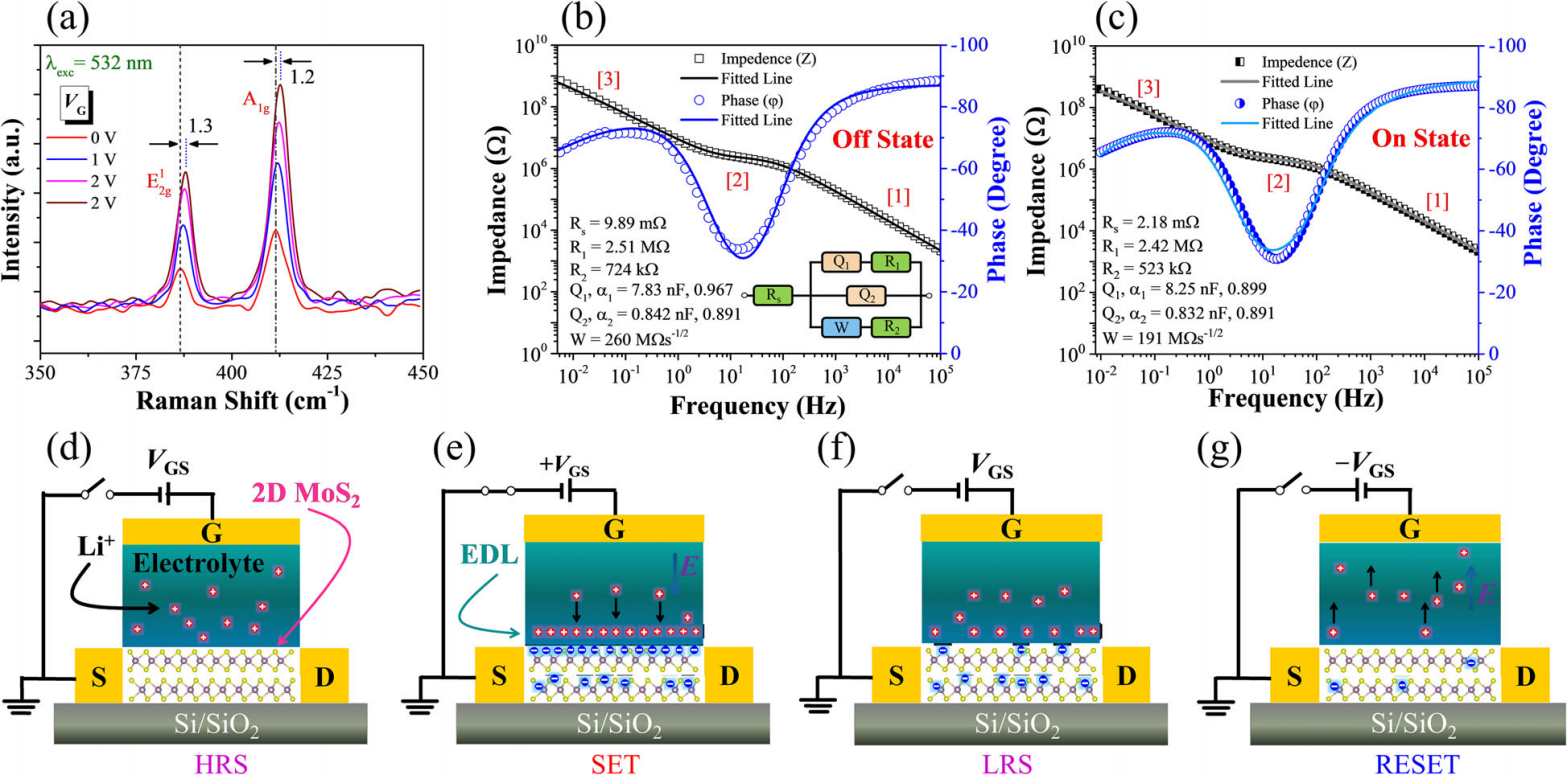
(a) Raman scattering spectra were recorded while applying different bias voltages at the gate. (b, c) Bode plots of the electrochemical impedance spectroscopy (EIS) data for the HRS and LRS, respectively, measured between the source‐gate (S‐G) terminals. The inset in (b) shows the corresponding R‐C equivalent circuit model. (d–g) Schematic illustration of coupled electron‐ion carrier dynamics governing device operation: (d) pristine state; (e) SET bias applied at the gate, leading to electric double layer (EDL) formation at the MoS_2_/electrolyte interface; (f) induced low‐resistance state (LRS) retained after removal of the SET bias; and (g) RESET bias at the gate, enabling erasure and restoration of the channel conductance to the pristine state.

### 2.3 Brain‐Inspired Synaptic Plasticity for Learning and Memory

The synergy of synapse with the 2D MoS_2_ IMT is illustrated schematically in Figure 3a, where the side gate serves as the presynaptic terminal and the drain current represents the postsynaptic response. Channel conductance encodes the synaptic weight. The excitatory post‐synaptic current (EPSC) response, a key indicator of brain‐like synaptic behavior (Figure S17), exhibits fast and slow relaxation times of approximately 5 and 670 ms, respectively, consistent with the values obtained from impedance analysis. The fast relaxation arises from ion accumulation and relaxation at the MoS_2_/electrolyte interface, while the slower relaxation is attributed to the gradual redistribution of Li^+^ ions within the solid polymer electrolyte after the pulse is removed. Paired‐pulse facilitation (PPF) and paired‐pulse depression (PPD) were emulated by applying identical voltage pairs at the gate (Figure 3b,c). A +2.0 V, 50 ms pulse pair induces PPF, whereas a −1.0 V, 250 ms pair produces PPD. The second pulse yields enhanced conductance in PPF and suppressed conductance in PPD, reflecting short‐term synaptic potentiation and depression. The PPF/PPD index, defined as [(A_2_ – A_1_)/A_1_] × 100%, decreases with increasing inter‐pulse interval (Δ*t*), consistent with time‐dependent Li^+^ redistribution at the MoS_2_/SPE interface. The PPF decay follows a double‐exponential function (τ_1_ = 24.7 ms, τ_2_ = 2001 ms), while PPD obeys a single‐exponential relaxation (τ_3_ = 95.1 ms), comparable to characteristic biological timescales (Figure 3d) [36]. By modulating pulse amplitude, width and number, the device further reproduces transitions from short‐lived sensory resister (SR) to persistent conductance states, short‐ and long‐term memory (STM and LTM), analogous to rehearsal‐driven memory consolidation as proposed in Atkinson‐Shiffrin memorization model (Figure 3e–g). Repeated stimulation progressively strengthens the conductance, mimicking activity‐dependent memory strengthening. Spike‐timing‐dependent plasticity (STDP) was evaluated by measuring the relative conductance change, ΔG = [(G – G_0_)/G_0_] × 100%, as a function of input spike timing at the gate (Figure 3h–k) [37]. Asymmetric Hebbian and anti‐Hebbian rules, together with their symmetric counterparts, were realized successfully. Potentiation or depression depends on the relative spike order and decays with increasing |Δ*t*|, following canonical STDP learning functions [38, 39]. Importantly, the asymmetric learning behavior shows excellent agreement with the STDP learning function, whereas the symmetric learning behavior is well described by Gaussian fitting (Note S7). The ability to implement all four STDP modes highlights the versatility of ion‐mediated conductance modulation for neuromorphic hardware.

**FIGURE 3:**
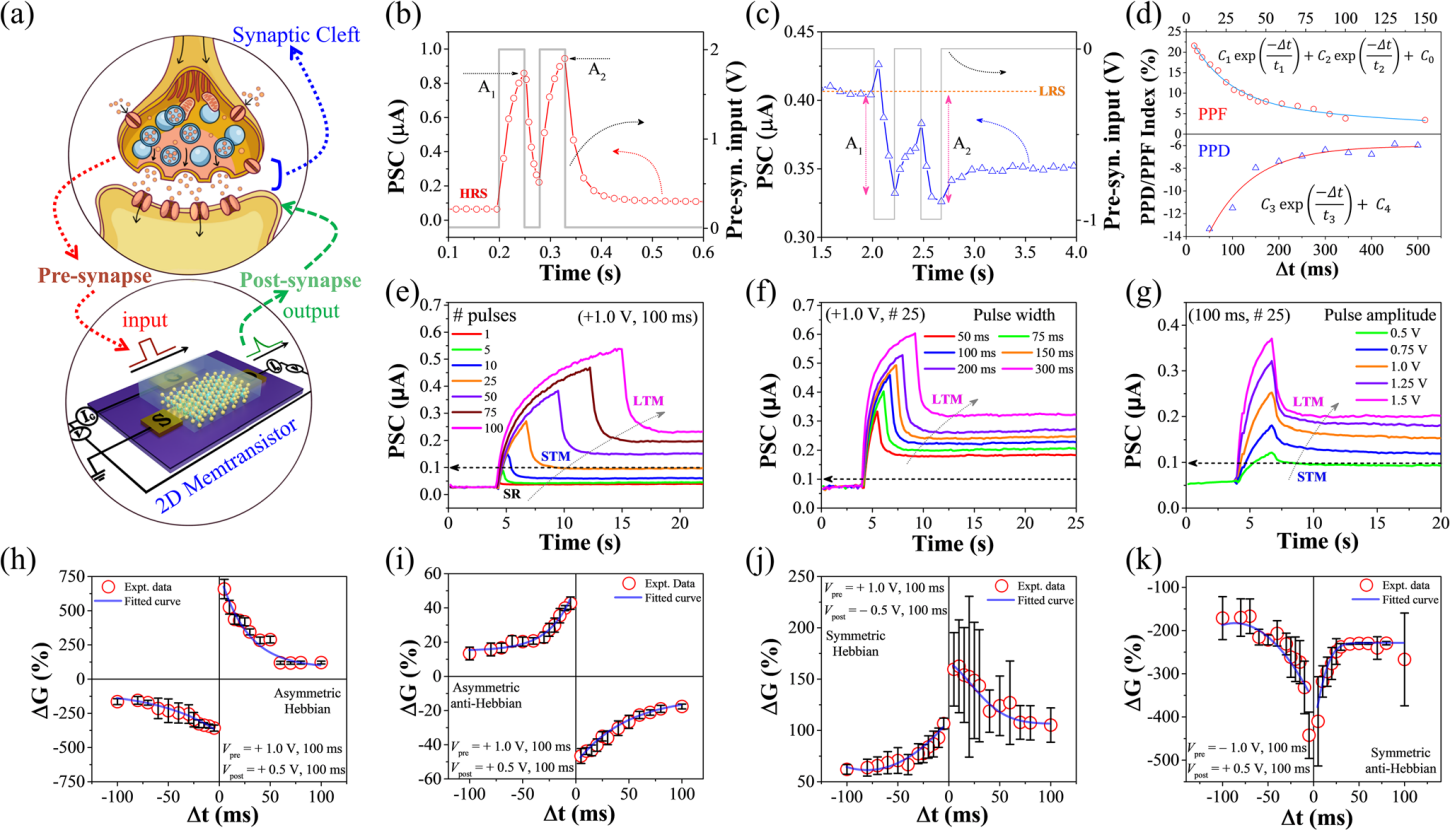
(a) Analogy between a biological synapse and the 2D MoS_2_ IMT device as synapse. (b, c) PPF and PPD responses, respectively, recorded as the postsynaptic current (PSC) upon application of a paired presynaptic voltage pulse (gray line). *A*_1_ and *A*_2_ represent the conductance corresponding to the first and second pulses. (d) PPF (top panel) and PPD (bottom panel) index plots. (e–g) Emulation of the Atkinson‐Shiffrin memory model through modulation of presynaptic input pulse parameters: pulse number (*n*), pulse width (*t*_w_), and pulse amplitude (*V*_m_). (e) Variation of *n* with *t*_w_ and *V*_m_ held constant. (f) Variation of *t*_w_ with *n* and *V*_m_ held constant. (g) Variation of *V*_m_ with *t*_w_ and *n* held constant. A gradual transition from sensory register (SR) to short‐term memory (STM) and long‐term memory (LTM) is observed, with a postsynaptic current (PSC) threshold of 100 nA defined as the STM‐LTM transition boundary. (h–k) Implementation of simplest unsupervised learning rules: (h) asymmetric Hebbian learning; (i) asymmetric anti‐Hebbian learning; (j) symmetric Hebbian learning; and (k) symmetric anti‐Hebbian learning.

### 2.4 Hardware‐Emulated Adaptive Learning via Repeated Rehearsal

Learning‐forgetting‐relearning is a fundamental adaptive process in biological systems, wherein prior training accelerates subsequent relearning. This behavior was emulated in the 2D IMT by applying presynaptic voltage pulses at the gate and monitoring the postsynaptic drain current (PSC). An increase in drain current under stimulation is defined as learning, whereas its spontaneous decay after pulse removal corresponds to forgetting. As shown in Figure 4a, 100 consecutive pulses (+1 V, 100 ms) raise the current to ∼1.6 µA during the initial training phase. After a 20 s relaxation period without stimulation, the conductance partially decays. Remarkably, only 25 pulses are required in the second training stage to restore the current to its previous level, demonstrating facilitated relearning enabled by residual interfacial Li^+^ accumulation. This adaptive behavior, typically emerging from complex neural networks, is reproduced here within a single 2D IMT device through intrinsic ion‐electron dynamics. Higher‐order associative learning was further demonstrated by emulating classical conditioning like Pavlov's dogs experiment. Distinct presynaptic pulse trains were assigned as neutral stimulus (NS, +0.5 V, 100 ms) and unconditioned stimulus (UCS, +1 V, 100 ms), while the postsynaptic pulse (*V*_D_ = +0.01 V) was used to record the PSC response (Figure 4b). Initially, the NS alone produces a subthreshold response as no salivation, whereas the UCS induces strong facilitation. Repeated paired presentation of NS and UCS progressively enhances the PSC response to NS alone, indicating the formation of stimulus association. After training, the NS elicits a supra‐threshold current (> 0.2 µA), analogous to a conditioned response (CR). This associative plasticity highlights the capability of iontronic modulation to reproduce adaptive learning functions in hardware.

**FIGURE 4:**
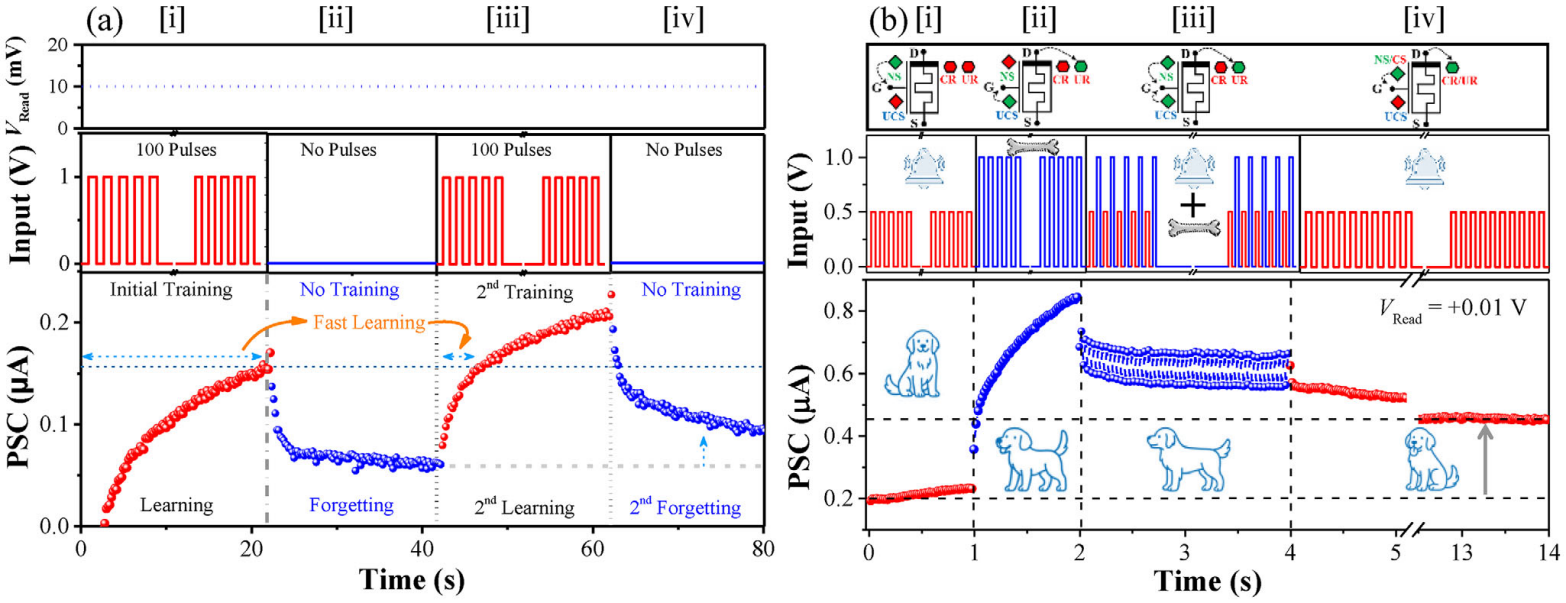
(a) Progressive learning and natural forgetting demonstrated through repetitive presynaptic stimulation. A train of 100 identical voltage pulses applied at the gate induces cumulative synaptic potentiation, reflected by a gradual increase in postsynaptic current (PSC) ([i] and [iii]). The intermediate regions ([ii] and [iv]) correspond to a 20 s relaxation interval without stimulation, during which the PSC partially decays due to spontaneous ion back‐diffusion, representing natural forgetting. (b) Emulation of classical conditioning using the 2D IMT. [i] A low‐amplitude pulse train applied at the gate as the neutral stimulus (NS; bell) produces a minimal, subthreshold PSC response at the drain. [ii] A higher‐amplitude pulse train assigned as the unconditioned stimulus (UCS; food) generates a pronounced PSC exceeding the defined threshold, corresponding to the unconditioned response (UR). [iii] During training, alternating NS and UCS pulses progressively enhance the synaptic weight, establishing stimulus association. [iv] After conditioning, the NS alone elicits a suprathreshold PSC, now functioning as a conditioned stimulus (CS) that produces a conditioned response (CR).

### 2.5 Pattern Classification Utilizing Measured Synaptic Weights

Long‐term potentiation (LTP) and long‐term depression (LTD), which enable persistent strengthening or weakening of synaptic weight under repeated stimulation, are central to hardware implementation of artificial neural networks [22, 40]. In the 2D IMT, synaptic weight updates were realized using controlled gate‐voltage stimuli. Potentiation/depression (P/D) characteristics were first obtained by applying stepwise gate pulses from 0 to +2.0 V (10 mV increments, 50 ms) and subsequently reversing the sequence (Figure S18a). Analogous channel conductance behavior as synaptic plasticity was confirmed using 1000 identical excitatory (+1.0 V, 1 ms) and inhibitory (−1.0 V, 1 ms) pulses (Figure S18b). The linearity and symmetry of conductance evolution, critical for reducing training error, were evaluated by fitting the weight‐update response to a standard synaptic learning model (Note S8), revealing near‐linear and well‐balanced P/D characteristics. For system‐level validation, experimentally measured conductance modulation was used to construct device‐specific STDP kernels, which informed the temporal encoding and weight initialization during network training [41, 42]. Figure 5a shows representative P/D curves recorded using 100 excitatory (+1.0 V, 10 ms) and inhibitory (‒1.0 V, 10 ms) pulses. The gradual analogue conductance states were mapped via cubic spline function‐based interpolation to ensure smooth integration into the training algorithm (Figure 5b). A hybrid spiking‐convolutional neural network (SNN/CNN) architecture was then constructed (Figure 5c) and trained using the MNIST handwritten‐digit dataset [43, 44]. The network achieved a recognition accuracy of ∼99.4% with a training loss below 2.5% after 25 epochs only (Figure 5d). Reconstructed output images and the confusion matrix (Figure 5e) exhibit strong diagonal dominance, indicating high classification fidelity and reliable hardware‐informed learning performance.

**FIGURE 5:**
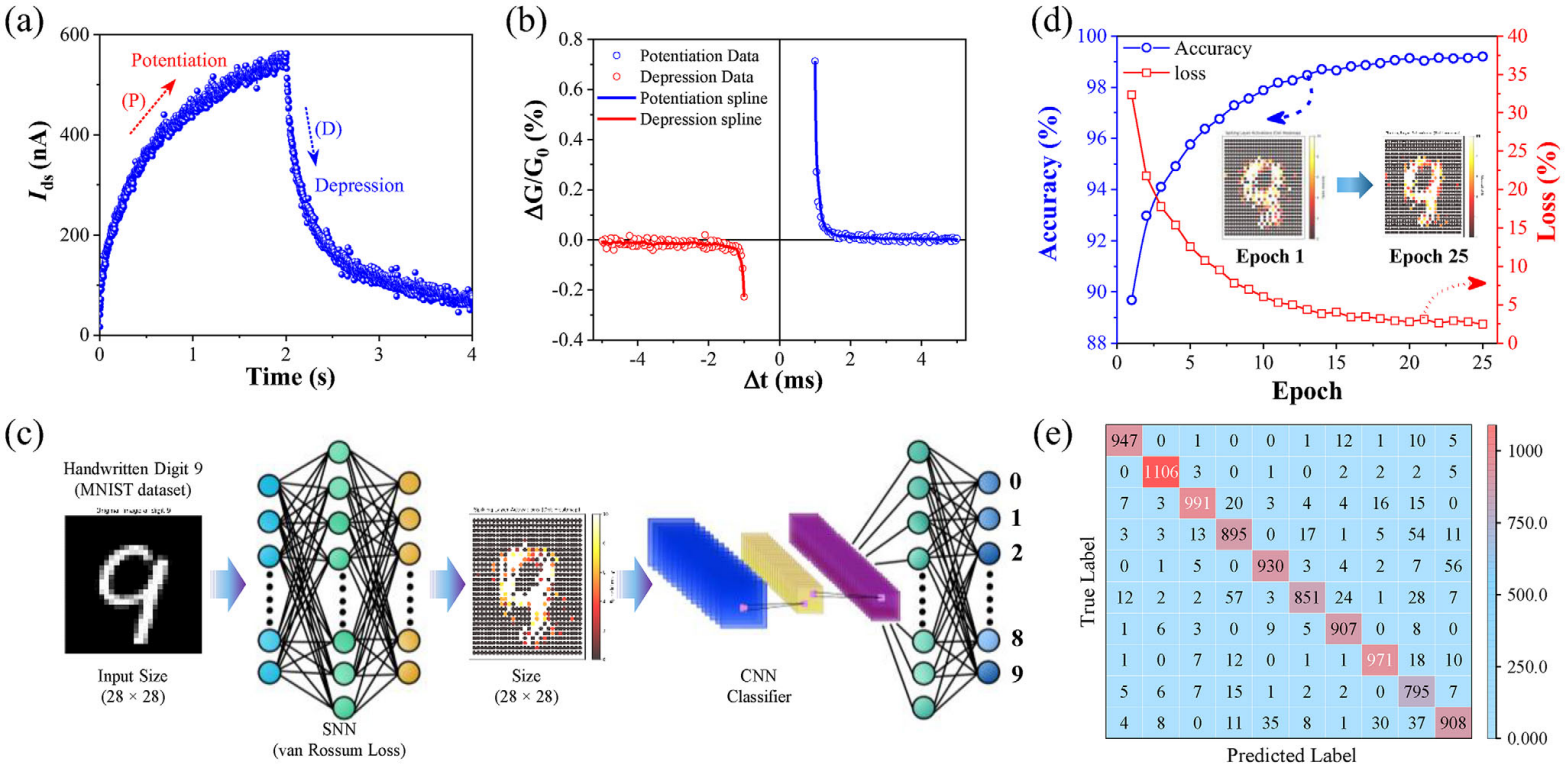
(a) Potentiation and depression (P‐D) characteristics of synaptic weight obtained under 100 consecutive excitatory and inhibitory voltage pulses, demonstrating gradual analogue conductance modulation. (b) Spike‐timing‐dependent plasticity (STDP) behavior derived from the measured P‐D characteristics. The solid line represents a cubic spline interpolation used to provide a smooth and continuous mapping for neural‐network training. (c) Schematic illustration of MNIST handwritten‐digit classification implemented using a hybrid spiking‐convolutional neural network (SNN/CNN) architecture incorporating experimentally extracted synaptic parameters. (d) Evolution of training loss (red squares) and classification accuracy (blue circles) as a function of training epochs. The inset shows reconstructed output images of the digit “9” after epoch 1 and epoch 25, indicating progressive learning. (e) Confusion matrix summarizing predicted versus true digit labels, demonstrating high classification fidelity with strong diagonal dominance.

## 3 Conclusions

We have demonstrated an iontronic 2D memtransistor that integrates electrochemical gating with analogue conductance modulation to emulate key synaptic and adaptive learning functions in hardware. In situ Raman spectroscopy and impedance analysis collectively confirm that device operation is governed by reversible Li^+^ accumulation at the MoS_2_/electrolyte interface rather than structural phase transformation, establishing electric double‐layer formation as the primary mechanism of conductance control. This interfacial ion‐electron coupling enables stable counter‐clockwise resistive switching, low variability across cycles and batches, and finely tunable multi‐level conductance states. Beyond device‐level characteristics, the 2D IMT reproduces biologically relevant plasticity, including short‐term facilitation and depression, long‐term potentiation/depression, spike‐timing‐dependent plasticity in all four canonical forms, and higher‐order associative learning. The facilitated relearning behavior and classical conditioning further highlight the intrinsic memory dynamics enabled by controlled ionic redistribution. Importantly, near‐linear and symmetric weight updates allow direct incorporation of experimentally measured conductance states into neural‐network simulations, yielding high recognition accuracy on standard benchmark tasks. These results bridge nanoscale iontronic physics with system‐level neuromorphic functionality. The present architecture offers several advantages: low‐voltage operation, material compatibility with scalable two‐dimensional semiconductors, and tunability through electrolyte composition. Future efforts may focus on reducing device variability at wafer scale, accelerating ion kinetics for high‐frequency operation, and integrating dense crossbar arrays for in‐memory computing. Extending this platform to heterogeneous 2D materials and solid‐state ion conductors could further enhance energy efficiency and retention. Collectively, our findings position iontronic 2D memtransistors as a promising route toward adaptive, hardware‐native neuromorphic systems that merge materials physics with cognitive functionality.

## 4 Experimental Methods

### 4.1 Mechanical Exfoliation of MoS_2_ Flakes

MoS_2_ flakes were mechanically exfoliated using scotch tape method on a Gel‐Pak elastomeric stamp. An optical microscope was used to localize the target MoS_2_ flake on the gel film. This MoS_2_ flake was transferred precisely onto the channel between the S‐D electrode via a custom‐made transfer stage, including micromanipulators and a long working distance optical microscope.

The MoS_2_ flakes were subjected to various characterizations before moving on to the device fabrication process. The material properties were recorded by Raman scattering spectroscopy and Photo Luminescence (PL) spectroscopy using a Horiba Scientific XploRA PLUS confocal Raman spectrometer with an excitation laser of 532 nm at room temperature. To record the Raman spectra, the excitation laser power was kept below 1 mW to avoid sample burning. The scattered emission was collected using a 50× objective and dispersed by an 1800 lines mm^−1^ grating. The PL spectra were recorded using a 50× objective, dispersed by a 600 lines mm^−1^ grating. The topography of the flake was characterized by a Park NX7 atomic force microscope (AFM) at room temperature in the tapping mode. For the scan, a set point of 0.7 V was considered using a cantilever with a force constant of 42 N m^−1^ and a resonant frequency of 330 kHz.

### 4.2 2D Iontronic Memtransistor Device Fabrication

The iontronic 2D memtransistor (IMT) devices were fabricated in a planar architecture, where source (S), drain (D), and gate (G) electrodes all lie in the same plane on the substrate. Briefly, the photo lithography technique was used to pattern the electrodes on a Si/SiO_2_ substrate. S‐D and G electrodes were formed on the substrate using standard photolithography and lift‐off process, followed by Cr/Au (10/50 nm) metal deposition. A 2D‐MoS_2_ flake was mechanically exfoliated on a gel‐film and transferred between the S‐D channel using a dry transfer technique. To prepare the electrolyte solution, PEO and LiClO_4_ were dissolved in anhydrous acetonitrile with two different w/w ratio of 9:1 and 4:1 to make a 4 wt.% solution. The mixture was then stirred overnight at 30^○^C. Finally, 1 µL of the prepared solution was then drop‐casted exactly on the junction of S‐D and G electrodes, and allowed to dry in a vacuum environment to complete the fabrication of the memtransistor device.

### 4.3 Electrical and Electrochemical Characterization

Electrical measurements were performed with a Keithley 4200 SCS parameter analyzer at room temperature under ambient conditions and in the dark. For the 3‐terminal transfer characteristic measurement, the voltage was swept at the gate (G) terminal, keeping the S and D terminals at constant bias, and the corresponding drain current (*I*_D_) was measured. To record the output characteristic, the S‐D voltage was swept and gate voltage (*V*_G_) was increased in steps. For the pulse based memory measurements, voltage pulses (*V*_G_) were applied to the G terminal using the Keithley 4200 SCS equipped with a PMU, and the corresponding drain current (*I*_D_) was measured at a constant S‐D bias.

For the ReRAM measurements, a write (W) voltage pulse of +2 V, 1 s was applied to the gate (G) terminal to switch the device to the low resistance state (LRS), followed by 5 read (R) pulses of +0.01 V, 100 ms at the drain (D) terminal to probe the current state. Then, an erase (E) voltage pulse of −2 V, 1 s was applied to the gate terminal to reset the device back to the high resistance state (HRS), followed by 5 read (R) pulses of +0.01 V, 100 ms at the drain (D) terminal to probe the current state to complete a W/5R/E/5R cycle. To record the retention of the device in multiple states, write pulses of +1 V, 100 ms was used at the gate terminal to change the conducting state of the device, and the current state was recorded by applying a constant read voltage bias of +0.01 V at an interval of 1 s at the drain terminal, finally an erase pulse of −1 V, 100 ms was applied to bring the device back to initial state.

We used two IMT devices connected in parallel and series to implement the two input logic ‘OR’ and ‘AND’ gate operations, respectively. The gate terminals were used as the input terminals to apply the voltage pulses. The two PMUs of the Keithley 4200‐SCS parameter analyzer were used to apply input pulses to the memtransistors, a Keysight B2987 source measuring unit (SMU) was used to apply the constant bias of +0.1 V at the drain terminal, and a Keithley 2602B SMU to measure the output current at the source terminal. Detailed discussion for the logic gate implementation is provided in Note S6.

The transient response was characterized by applying a +1.0 V gate‐voltage pulse of width of 5 ms while maintaining a bias of 0.05 V applied at the drain. The drain current was continuously monitored during the pulse application. The response time is defined as the time required for the drain current to rise from 10% to 90% of its steady‐state value following the application of the input gate pulse. Under the present measurement conditions, the device exhibited a response time of approximately 1 µs.

A Zahner Zennium Pro electrochemical workstation procured from Zahner‐Elektrik GmbH & Co. KG, Germany, was used to record the electrochemical impedance spectroscopy (EIS) data to study the dielectric properties of the solid electrolyte PEO: LiClO_4_. The frequency was swept from 1 MHz to 10 mHz with an alternating voltage of amplitude set at 50 mV to record the impedance data using the THALES XT Software Suite. The impedance results were analyzed using the Zahner analysis software for fitting and modeling to extract the RC equivalent circuit.

### 4.4 Electrical Programming for Synaptic and Cognitive Emulation

For all the synaptic measurements, the gate terminal was considered as the presynaptic terminal and drain terminal as the postsynaptic terminal. A Keithley 4200‐SCS parameter analyzer with a pulse measuring unit (PMU) was utilized to apply the necessary pulses to the gate terminal and measure the current at the S‐D terminal. For the paired pulse facilitation (PPF) measurements, a pair of identical voltage pulses were applied to the presynaptic terminal, and the response was recorded at the postsynaptic terminal using +0.01 V voltage bias. To get the PPF index, the time gap between the two presynaptic pulses was varied from 5 to 150 ms. For the paired pulse depression (PPD), first the device was taken to the ON state using +1 V, 100 ms pulse at the presynaptic terminal. Then, a pair of identical presynaptic pulses of (−1 V, 250 ms) were applied, and the corresponding postsynaptic current were recorded with +0.01 V drain bias. To get the PPD index, the time gap between the paired pulses was varied from 50 to 500 ms.

For the plasticity measurements, identical voltage pulses were applied at the presynaptic terminal and then the postsynaptic current was recorded using a read voltage bias of + 0.01 V at the postsynaptic terminal. During the measurement, one of the parameters such as number of pulses, pulse width and pulse amplitude were varied, keeping other two constant.

To demonstrate spike‐time‐dependent plasticity (STDP) measurements, both the pre‐ and postsynaptic pulses were applied using Keithley 4200‐SCS. The corresponding response is recorded by applying a postsynaptic bias of +0.01 V at the drain terminal. For each type of the STDP measurement, five independent sets of data points were acquired and the averaged results were plotted with error bars to indicate variability (details in Note S7).

To emulate synaptic plasticity by recording the potentiation and depression of the conducting states, potentiating pulses of +1 V, 1 ms, and depressing pulses of −1 V, 1 ms were applied to the presynaptic terminal, and the change in the conducting state was recorded by applying a read bias of +0.01 V at the postsynaptic terminal. In addition, a step like pulse scheme was employed to emulate the synaptic weight modulation. For potentiation, the gate voltage was increased from 0 to +2 V with a step size of 10 mV. Conversely, the gate voltage was decreased from +2 to 0 V with a 10 mV step size. The change in the conducting states were recorded by applying a read bias of +0.01 V at the postsynaptic terminal.

For the learning‐forgetting‐relearning process demonstration, the input voltage pulse train was applied to the presynaptic terminal and the postsynaptic current (PSC) response was recorded at the post synaptic terminal by applying a read voltage of +0.01 V. To demonstrate adaptive learning, we performed the experiments analogously to Pavlov's dog experiment. The input pulses were applied to the presynaptic terminal and the postsynaptic current (PSC) response was recorded at the post synaptic terminal by applying a read voltage of +0.01 V.

### 4.5 Neural‐Network Modeling and Pattern‐Recognition Simulations

For the neural network based pattern recognition study, the experimentally measured potentiation/depression (P/D) characteristics of the 2D MoS_2_ IMT device and generated spike‐timing dependent plasticity (STDP) characteristics were used as the fundamental training Kernels. The conductance updates under consecutive voltage pulses were extracted and normalized, followed by the generation of STDP data and cubic spline interpolation to generate smooth fitting curves suitable for large scale simulation. These device derived learning rules were then integrated into a hybrid spiking convolutional neural network (SNN/CNN) model designed for handwritten digit classification tasks. Training was carried out for 25 epochs using a benchmark dataset, where the memristor weights were initialized based on measured conductance windows and dynamically updated according to the spline fitted STDP response. The training and validation accuracy, as well as the van Rossum loss, were monitored throughout, with results demonstrating stable convergence and minimal overfitting. Output digit reconstructions and confusion matrix analyses were employed to further validate classification accuracy and loss distribution. Together, these methods establish a direct link between experimentally validated device characteristics and high‐fidelity cognitive computing tasks.

## Author Contributions

**Bikas C. Das**: conceptualization, investigation, formal analysis, validation, supervision, funding acquisition, writing – original draft, writing – review and editing. **Saptarshi Bej**: validation, methodology, software, writing – review and editing. **Puranjay Saha**: methodology, data curation, formal analysis, validation, writing – review and editing.

## Conflicts of Interest

The authors declare no conflicts of interest.

## Supporting information




**Supporting File**: advs77150‐sup‐0001‐S2_EGT‐R1.docx.

## Data Availability

The data that support the findings of this study are available from the corresponding author upon reasonable request.
