# Supplementary material for: Analog Synaptic Plasticity in 2D Layered Material Iontronic Memtransistors for Brain‐Inspired Computing
Source: Adv Sci (Weinh). 2026 Aug 18;13(62):e77150. doi: 10.1002/advs.77150 (PMC13482939; doi:10.1002/advs.77150)
Supplement: Supplementary file 1 [file ADVS-13-e77150-s001.docx]

Supporting Information

Analog Synaptic Plasticity in 2D Layered Material Iontronic Memtransistors for Brain-Inspired Computing

Puranjay Saha,^1^ Saptarshi Bej,^2^ and Bikas C. Das^1,^*

^1^ eNDR Laboratory, School of Physics, IISER Thiruvananthapuram, Trivandrum, Kerala 695551, India

^2^ School of Data Science, IISER Thiruvananthapuram, Trivandrum, Kerala 695551, India

* Corresponding Author

Email: [bikas@iisertvm.ac.in](mailto:bikas@iisertvm.ac.in)

ORCID ID:

Bikas C. Das: 0000-0002-4750-0542

**This file includes:**

Supporting Information Notes

Figs. S1 to S18

Tables S1

**Fig. S1.** AFM topography images of different exfoliated 2D-MoS_2_ flakes used to fabricate different devices. The inset shows the height profile of the flakes along the blue arrow line.

**Fig. S2.** Material characterization of the 2D MoS_2_. **a.** Raman scattering spectrum of 2D MoS_2_ flake on a SiO_2_ substrate with distinct characteristic peaks for in-plane $E_{2g}^{1}$ and out of plane $A_{1g}$ vibrational modes at 385.51 cm^−1^ and 404.79 cm^−1^, with a wavenumber difference (Δ) of 19.28 cm^−1^. **b.** PL spectrum of 2D MoS_2_ flake on a SiO_2_ substrate show a strong A-excitonic peak at 1.88 eV, confirming the direct bandgap of the exfoliated flake.

**Fig. S3.** **a.** Optical image of the fabricated 2D MoS_2_ IMT device. **b.** Magnified image of the 2D MoS_2_ flake on the S-D channel.

**Supporting Information Note 1: Two terminal *I-V* Characterizations**

Fig. S4a,b shows the schematic of the device and electrical connections for the *I-V* characterization of the S-D and S-G terminals before and after dropcasting the solid electrolyte. S-D channel has a length of 10 μm, and S-G has a channel length of 100 μm. Fig. S4c,d shows the *I-V* characteristics at the S-D channel, before and after dropcasting the electrolyte, respectively. A hysteresis-free *I-V* curve in Fig. S4c shows the semiconducting nature of the 2D MoS_2_ at the S-D terminal without the electrolyte. After the electrolyte was dropcasted, a small hysteresis arises, attributed to ionic motion in the solid electrolyte (Fig. S4d). Fig. S4e,f shows the *I-V* characteristics at the S-G channel, before and after dropcasting the electrolyte. Without the electrolyte, the *I-V* curve shows a pure dielectric nature. With the electrolyte, the current increased from pA to nA range, due to the ionic motions in the solid electrolyte.

**Fig. S4.** Two terminal *I-V* characterization of the IMT device at different fabrication stages. **a,b.** Schematic of the fabricated device and electrical connection with and without the electrolyte, respectively. **c,d.** The *I-V* characteristics at the S-D channel before and after dropcasting the electrolyte, respectively. The inset shows the optical image of the device. **e,f.** The *I-V* characteristics at the S-G channel before and after dropcasting the electrolyte, respectively.

**Supporting Information Note 2: Calculation of Field Effect Mobility (**$\boldsymbol{\mu}_{\mathbf{FE}}\boldsymbol{)}$ **_,_Threshold Voltage (**$\boldsymbol{V}_{\mathbf{TH}}\boldsymbol{)}$***_,_* Transconductance (**$\boldsymbol{g}_{\mathbf{m}}$**)**

The field effect mobility was calculated in the linear regime, using the following equation.

$\mu_{\mathrm{FE}}=g_{m}\times\frac{L}{W\times C_{i}\times V_{D}}$	(1)

Where channel length (*L*) = 10 μm, channel width (*W*) = 50 μm, *V*_D_ is the constant drain bias = +0.05 V. $C_{i}$ = 0.17 × 10^-3^ F/cm^2^ is the capacitance per unit area calculated from the recorded electrochemical impedance spectroscopy (EIS) data. The *V*_TH_ was calculated using extrapolation in the linear region (ELR) method shown in Fig. S5b. $g_{m}$ is the transconductance calculated from the transfer curve, defined as, $g_{m}= d(I_{D})/d(V_{G})$, shown in Fig. S5c.

**Fig. S5.** Transistor parameters extraction. **a.** Transfer curve used to calculate threshold voltage (*V*_TH_), transconductance ($g_{m}$) and field effect mobility ($\mu_{FE}$). **b.** Threshold voltage (*V*_TH_) calculated using the extrapolation in the linear region (ELR) method_._ **c.** Transconductance ($g_{m}$) calculated from the transfer curve. **d.** Field effect mobility ($\mu_{FE}$) calculated from the forward sweep of the transfer curve.

Supporting Information Note 3: Statistical Analysis of Cycle-to-Cycle and Device-to-Device Performance Variation

The statistical distributions of the key device parameters, namely threshold voltage (*V*_TH_), subthreshold swing (SS) are presented in Fig. S7a, d. The histogram with normal distribution fit for *V*_TH_ (Fig. S7a) shows a narrow distribution with a mean value of 0.828 V and a standard deviation of only 3.6 mV, highlighting the excellent cycle-to-cycle uniformity. The SS distribution (Fig. S7b) exhibits an average of 416.1 mV/dec with a standard deviation of 47 mV/dec. Furthermore, the device-to-device variation depicted in Fig. S7c, d confirms that the high stability and uniformity of *V*_TH_ and SS is maintained across multiple fabricated devices.

The cumulative probability plot of the currents in high resistance state (HRS) and low resistance state (LRS) shows a seperation of ~10^4^ between the two states (Fig. S8a). The Weibull distribution (Fig. S8b) provides a slope factor of 35.5 and a characteristic value of 9.9 × 10^−6^ A for the LRS current and 10.6 slope factor and a characteristic value of 2.7 × 10^−9^ A for the HRS current. Further the cumulative probability and Weibull distribution of the LRS and HRS currents for different devices confirm high stability and uniformity across different fabricated devices (Fig. S8c, d).

**Fig. S6.** Transfer characteristics of the fabricated IMT devices in different batches. The transfer curves were recorded by sweeping the *V*_G_ from −1.0 V to +2.0 V at a constant *V*_D_ = +0.05 V (red line), and symultaneously recording The gate leakage current (blue dashed line).

**Fig. S7.** Statistical analysis of cycle-to-cycle and device-to-device variation. **a,b.** Histogram with normal distribution of the threshold voltage (*V*_TH_) and subthreshold swing (*SS*) extracted from the 300 transfer curves in Figure 1e. **c,d.** Box plot with normal distribution of threshold voltage (*V*_TH_) and subthreshold swing (SS), extracted from the transfer curves of the six different devices in Fig. S6.

**Fig. S8.** **a,b.** Cumulative probability distribution and Weibull distribution of the LRS and HRS, extracted from the 300 transfer curves in Figure 1e. **c,d.** Statistical cumulative and Weibull distribution of LRS and HRS currents for the devices fabricated in different batches, respectively.

**Supporting Information Note 4: Electrical Characterization of IMT Devices Fabricated with 1:4 LiClO_4_: PEO Weight Ratio**

A LiClO_4_:PEO electrolyte solution (4 wt%) with a w/w ratio 1:4 was prepared and used to fabricate the IMT device. The same MoS_2_ flake shown in Fig. S3, was used as the channel material. The device had a channel width of 500 μm and channel length 10 μm. After the fabrication, *I-V* characteristics were recorded across the S-D and S-G terminals (Fig. S9a,b). Subsequently, the transfer characteristics of the device was recorded by sweeping *V*_G_ from –1 V to +2 V and back to –1 V at a fixed drain voltage bias *V*_D_ = +0.05 V (red curve), simultaneously recording the gate current *I*_G_ (blue curve). Consecutive transfer curves were recorded over 250 cycles, which shows excellent stability and low leakage current (Fig. S9c). It should be noted that the average memory window for the devices with 1:4 ratio of LiCLO_4_:PEO was found to be ~0.62 V, which is smaller than that observed for devices made with 1:9 ratio of LiClO_4_ and PEO (~0.9 V). This reduction in memory window is attributed to the higher electrolyte concentration, which also results in increased gate leakage current. The output characteristics of the device were recorded by sweeping the *V*_D_ from −0.1 V to +0.5 V for each applied *V*_G_ varying from 0 to +3 V in steps of 100 mV, as shown in Fig. S9d. Bias stress measurements were performed to evaluate the stability of the fabricated IMT device. A constant gate voltage (*V*_G_) bias of +1.0 V was applied, and the transfer characteristics were recorded at *V*_D_ = +0.05 V over different time intervals. Fig. S9e presents the transfer curves obtained during the bias stress test. The extracted *V*_TH_ for each transfer curve is shown in the Fig. S9f.

**Fig. S9.** Electrical Characteristics of the fabricated 2D IMT device using different electrolyte concentration. **a,b.** The *I-V* characteristics measured at the S-D and S-G terminals, respectively, after dropcasting the electrolyte. The inset shows the schematic of the fabricated device and electrical connection for the measurement. **c.** Transfer Characteristics (*I*_D_ vs *V*_G_, red line) recorded for 250 consecutive cycles with the corresponding leakage current (*I*_G_ vs *V*_G_, blue line). **d.** Output characteristics (*I*_D_ vs *V*_D_) recorded by sweeping *V*_D_ between –0.1 V and +0.5 V and varying *V*_G_ from 0 to +3 V in steps of 100 mV. **e.** Transfer characteristics recorded at regular time intervals during the positive bias stress (PBS) test over a period of 10^4^ s. **f.** Extracted threshold voltage (*V*_TH_) during the PBS stability test.

**Fig. S10.** **a.** Linear plot of the recorded transfer curves by varying *V*_D_ from +0.01 V to +0.1 V and sweeping *V*_G_ from −1.0 V to +2.0 V. **b.** Linear plot of the Transfer curve by varying the maximum *V*_G_ from +1.0 V to +3.0 V at constant *V*_D_ = +0.05 V.

**Supporting Information note 5: Signal-to-Noise Ratio (SNR) Calculation**

By computing the mean currents and standard deviation (SD) of the two consecutive memory states from the retention measurements, the signal-to-noise ratio (SNR) was determined. For the states to be distinguishable, the difference between the mean currents should be greater than the sum of their SDs. Hence, the SNR is defined as,

$SNR= \frac{I_{n+1}-I_{n}}{\sigma_{n+1}+\sigma_{n}}$	(2)

Where, $I_{n}$ and $I_{n+1}$ are the mean current of the nth and (n+1)th state, respectively. $\sigma_{n}$ and $\sigma_{n+1}$ are the standard deviation of the nth and (n+1)th state, respectively. To demonstrate the SNR calculation, consider the retention data of the 10^th^ and 11^th^ states as shown in Fig. S11b. The mean currents of the 10^th^ and 11^th^ states are 70.1 nA and 75.7 nA, respectively, with SDs of 1.05 nA and 1.25 nA, respectively. Using the mean and SD values for the 10^th^ and 11^th^ states, the resultant ratio is calculated to be 2.47, indicating the distinguishability of the two states as the ratio is greater than 1. Similarly, the SNR was calculated for all memory states. All computed ratios surpass the critical limit of SNR = 1, confirming the credibility of all the storage states.

**Fig. S11. a.** Pulse scheme used to record the multiple state retention. **b.** Drain currents measured during the retention test for the 10^th^ and 11^th^ states.

**Fig. S12.** Temporal drain current response measured at *V*_D_ = +0.05 V by applying a voltage pulse of +1.0 V, 5 ms at the gate.

**Supporting Information Note 6: Logic Gate Operations Using Iontronic 2D Memtransistors**

The Fig. S13a illustrates the circuit diagram for implementing the OR gate and the constructed truth table. Two of the fabricated IMT devices were connected in a parallel combination. The side gates of each memtransistor were used as the input terminals to apply inout voltage pulses (*IN*_1_ and *IN*_2_), and a constant voltage bias (*V*_bias_) of +0.1 V was applied to the drain terminal, and the output current was recorded at the source terminal. We have considered a voltage pulse of +2 V, 1s as the ‘1’ and 0 V as the ‘0’ for the input signals *V*_G1_ (in black) and *V*_G2_ (in red). The corresponding current (*I*_Out_) at the output terminal was recorded to demonstrate the OR gate operation, as shown in the Fig. S13a. A threshold current of 0.25 μA was chosen to distinguish between the ‘0’ and ‘1’ states. The output shows high current (‘1’) when any of the inputs is high (‘1’) and low current (‘0’) when both the inputs are low (‘0’), similar to an OR gate truth table.

Similarly, two IMT devices were connected in series to demonstrate AND gate operation as illustrated in the Fig. S13b. Here also, +2 V, 1s voltage pulse was considered high input (‘1’) and 0 V as the low input (‘0’). The ‘0’ and ‘1’ states of the output current (*I*_Out_) were classified as < 0.75 μA and > 0.75 μA, respectively. The output shows low current (‘0’) when any of the inputs is low (‘0’) and high current (‘1’) when both the inputs are high (‘1’), similar to an AND gate truth table. Interestingly, when we applied 10 pulses, the current showed a potentiating nature, confirming the device memory property.

**Fig. S13.** **a,b.** Demonstration of logic gate operation by reproducing the truth table of OR and AND gates, respectively, by applying voltage pulses at the gate terminal and recording the output current at the drain terminal. Each panel consists of the logic gate circuit, a truth table, and a plot of two input voltage pulse schemes with respective read current at the output.

**Fig. S14.** Raman spectra of 2D-MoS_2_ IMT device while applying voltage bias at the gate terminal. **a.** The full range shows the absence of a Raman peak around 200 cm^-1^, indicating no 1T’ phase formation. **b.** Enlarged view of **a** near the $E_{2g}^{1}$ and $A_{1g}$ peaks to highlight the peak intensity enhancement under higher positive gate bias voltage.

**Fig. S15.** Electrochemical impedance spectroscopy of the IMT device. **a.** Device schematic and the electrical connection for the EIS measurement between the S-G terminals. **b,c.** Nyquist plot for HRS and LRS respectively. Inset shows the equivalent circuit diagram used for the fitting.

**Fig. S16.** **a.** The frequency response of dielectric constant (ε’) and dielectric loss (tanδ). **b.** Capacitance vs frequency (*C* vs *f*) extracted from the recorded EIS data between the S-G terminal.

**Fig. S17.** Excitatory post-synaptic current (EPSC) response recorded by applying a voltage pulse (+1.0 V, 50 ms).

**Supporting Information note 7: Spike Time Dependent Plasticity (STDP) Learning Function**

The change of synaptic weight for asymmetric Hebbian (AHL) and anti-Hebbian learning (AAHL) rules can be fitted by an STDP learning function:

$\Delta G=A_{+}\exp\left( \frac{-\Delta t}{\tau_{+}} \right)+\Delta G_{0} (\Delta t>0)$	(3)

$\Delta G=A_{-}\exp\left( \frac{-\Delta t}{\tau_{-}} \right)+\Delta G_{0}(\Delta t<0)$	(4)

Where $\Delta G$ is the percentage change in synaptic weight, A is the scaling factor, $\Delta t$ is the pre/post spike time difference, $\tau$ is the time constant of the exponential function, and $\Delta G_{0}$ is a constant. The experimental data shown in Figure 4h, i are well described by the function in equations (3, 4). For the AHL in Figure 4h, $A_{+}$= 715.38, $A_{-}$= −306.4; $\tau_{+}$ = 27.53 ms and $\tau_{-}$ = 58.77 ms. For the AAHL in Figure 4i, $A_{+}$= 37.42, $A_{-}$= −39.8; $\tau_{+}$ = 22.24 ms and $\tau_{-}$ = 48.1 ms.

The change of synaptic weight for symmetric Hebbian (SHL) and anti-Hebbian learning (ASHL) rules shown in Figure 4j, k was fitted with a Gaussian function:

$\Delta G=A\exp\left( \frac{-{\Delta t}^{2}}{\omega^{2}} \right)+\Delta G_{0}$	(5)

Where $\Delta G$ is the percentage change in synaptic weight, A is the scaling factor, $\Delta t$ is the pre/post spike time difference, $\omega$ is the width of the timing window, and $\Delta G_{0}$ is a constant.

**Fig. S18.** **a.** Potentiation (P) and deprerssion (D) curves recorded by using step like voltage pulses. **b.** P/D curves recorded by applying identical pulse train. The insets show the applied pulse scheme for recording the P/D curves respectively.

Supporting Information Note 8: Nonlinearity, Symmetry, and Write Noise Analysis of the LTP/LTD curves

**Nonlinearity**

The nonlinearity of the long term potentiation (LTP) and long term depression (LTD) curves recorded in Supporting Information Fig S18a, b was obtained by fitting the curves with the following weight update equations:

$G_{n+1}=G_{n}+{\Delta G}_{P}=G_{n}+\alpha_{P}\exp(-\beta_{P}\frac{G_{n}-G_{min}}{G_{max}-G_{min}})$ (for LTP)	(6)

$G_{n+1}=G_{n}+{\Delta G}_{D}=G_{n}-\alpha_{D}\exp(-\beta_{D}\frac{G_{max}-G_{n}}{G_{max}-G_{min}})$ (for LTD)	(7)

where the $G_{n+1}$ and $G_{n}$ denote the synaptic conductance of the device when the $(n+1)$th and $n$th pulses were applied, and the parameters α and β are the changing step sizes of the conductance and nonlinearity, respectively.$G_{max}$ and $G_{min}$ denote the maximum and minimum consuctance state in the potentiation or depression curve, respectively. A larger value of β presents a greater nonlinearity, and for an ideal synaptic device, its weight updating processes should be linear (β = 0). Supporting Information Fig S19a,b illustrates the fitted weight update curves obtained from plotting $G_{n+1}$ vs $G_{n}$ of the LTP/LTD recorded using step like pulses. Supporting Information Fig S19d,e shows the weight update curves for the LTP/LTD recorded using voltage pulse train. The extracted parameters are tabulated in Table S1.

**Symmetricity**

The symmetricity is defined as the reciprocal of the symmetric error, *i.e.*, symmetricity = 1/symmetric error. The following equation can calculate the symmetric error:

$Symmetric error= \sum_{k=1}^{k=n} \frac{{{(G}_{N}\left( k \right)-G_{N}\left( 2n-k \right))}^{2}}{n}$

$=\frac{({G\left( k \right)-G\left( 2n-k \right))}^{2}}{n(G_{max}-{G_{min})}^{2}} where, G_{N}\left( k \right)= \frac{G\left( k \right)-G_{min}}{G_{max}-G_{min}}$	(8)

Here $G_{N}(k)$ represents the normalized conductance in the kth state, $G\left( k \right)$ represents the conductance in the k^th^ state. The symmetric error and symmetricity calculated using equation 8 are presented in Table S1.

**Write Noise**

The write noise is defined as the standard deviation of the measured channel conductance during multiple cycles. The calculated write noise for the LTP and LTD curves is presented in Table S1.

**Fig. S19.** Nonlinearity analysis of synaptic weight modulation. **a, b.** Nonlinearity (NL) analysis of the long-term potentiation (LTP) and long-term depression (LTD) characteristics obtained using step-like voltage pulses. **c.** Illustration highlighting the conductance of the k^th^ state in the potentiation branch and the corresponding (2n − k)^th^ state in the depression branch for step-like pulse operation. **d, e.** NL analysis of the LTP and LTD characteristics recorded using a voltage pulse train. **f.** Illustration highlighting the conductance of the k^th^ state in the potentiation branch and the corresponding (2n − k)^th^ state in the depression branch under pulse-train stimulation.

**Table S1. Extracted fitting parameters for the LTP/LTD curves of the 2D MoS_2_ IMT device**

|  | α_P_ (μS) | β_P_ | α_D_ (μS) | β_D_ | Symmetricity | Write noise |
| --- | --- | --- | --- | --- | --- | --- |
| Steplike Pulses | 0.15 | − 0.27 | 0.22 | 0.44 | 303.6 | 34.11% |
| Pulse train | 1.79 | 6.79 | 1.57 | 4.49 | 50.96 | 31.33% |
